# The Use of Dried Blood Spots for the Quantification of Antihypertensive Drugs

**DOI:** 10.1155/2018/3235072

**Published:** 2018-08-01

**Authors:** Alexander Chernonosov

**Affiliations:** Institute of Chemical Biology and Fundamental Medicine, Siberian Branch of Russian Academy of Sciences, Academician Lavrentiev Avenue 8, Novosibirsk 630090, Russia

## Abstract

Hypertension or high blood pressure is a harbinger of cardiovascular diseases. There are several classes of drugs used to treat hypertension. This review discusses the use of dried blood spots (DBSs) for the quantification by mass spectrometry (MS), tandem mass spectrometry (MS/MS), or, in some cases, by fluorescence detection methods the following antihypertensive medications: angiotensin-converting enzyme inhibitors (ramipril, ramiprilat, captopril, and lisinopril); angiotensin II receptor antagonists (valsartan, irbesartan, losartan, and losartan carboxylic acid); calcium channel blockers (verapamil, amlodipine, nifedipine, pregabalin, and diltiazem); *α* blockers (guanfacine, doxazosin, and prazosin); *β* blockers (propranolol, bisoprolol, atenolol, and metoprolol); endothelin receptor antagonists (bosentan and ambrisentan); and statins (simvastatin, atorvastatin, and rosuvastatin).

## 1. Introduction

Cardiovascular disease is the number one cause of death in the world [1]. Hypertension is the main attributable risk factor of death and is responsible for approximately half of the cases of cerebrovascular and ischemic heart diseases [2, 3]. Moreover, blood pressure over 140/90 mmHg is a cause of heart failure and chronic kidney disease in 20% and 23% of cases, respectively [3–5]. As a rule, high blood pressure is widespread among the elderly [3]. Blood pressure above 140/90 mmHg is observed in more than 50% of people aged 60 years and 75% over the age of 70 years [6, 7], and the treatment of hypertension is becoming an increasingly urgent problem with the ageing of society [3]. On the other hand, blood pressure >140/90, despite treatment with a diuretic and two other antihypertensive drugs of various classes, is defined as drug-resistant hypertension [8, 9]. In the USA, patients who need to take more than four antihypertensive drugs to achieve a normal blood pressure level are also considered resistant hypertensives [10].

Nevertheless, most people with hypertension require more than one drug for effective control of the disease. Because hypertension can develop in different biochemical ways, various classes of antihypertensive drugs have been developed. Diuretics, calcium channel blockers (CCBs), *α* blockers, *β* blockers (BBs), and inhibitors of the renin-angiotensin system are used for initial antihypertension therapy [11]. In addition, angiotensin-converting enzyme (ACE) inhibitors, angiotensin II receptor antagonists (ARAs), and endothelin receptor antagonists (ERAs) have been used for antihypertensive therapy over the past 20 years [12, 13]. CCBs, ARAs, and BBs have been the most prescribed antihypertensive medications [14–16]. Treatment of most patients (67.92 %) includes more than one drug. The most commonly used combination of medications has been CCB + BB + *α* blocker (7.55 %) [13]. For effective treatment, patients should follow the indications for taking a given drug and the prescribed dose [17]. The big problem is that ~50% of all patients with heart disease do not adhere to their prescribed regimen [18–20]. Often, such patients are referred to as patients with apparently resistant hypertension [9]. Patients, who ignore or do not adhere to their prescribed medication [21], more often as a result show considerable morbidity, higher costs of care, and mortality [22–26]. Many clinicians fail to assess blood pressure regularly or effectively titrate and regulate the dose of drugs; these shortcomings result in ineffective treatment [27].

The use of dried blood spots (DBSs) can simplify the methods for determining the concentrations of drugs. The DBS sampling technique is minimally invasive, and capillary blood can be obtained from a finger prick with a lancet by the patients themselves or guardians with minimal training. Such a sampling technique is also suitable even for small children [28] and is ideal for routine clinical testing [29] or helps with recruitment of subjects for preclinical or clinical studies [30]. Besides, DBSs reduce to a minimum the risk of infection with HIV and other infectious pathogens [31]. Moreover, DBSs offer a simpler storage and easier transfer by mail to the assigned laboratory, preventing unnecessary costs [32–34]. They should be well desiccated after sampling (2–3 hours minimum). The combined advantage of the above benefits coupled with improved analytical instrumental capability [35] has been recognized for the use of this methodology for various applications including therapeutic drug monitoring [36–38], toxicokinetic studies [39], and preclinical or clinical pharmacokinetic studies [30, 39–43].

In the current paper, quantification of major classes of antihypertensive drugs and statins in DBS by liquid chromatography with mass spectrometry (LC-MS), liquid chromatography with tandem mass spectrometry (LC-MS/MS), and fluorescence detection methods in recent years are reviewed. Parameters of analysis that can influence sensitivity were analyzed: types of used DBS cards, mass-spectrometers and detection modes, and the diameter of a disk punched for analysis, linear concentration range, elution solvents, extraction procedures, recovery, and stability of DBS samples.

## 2. DBS Analysis

The technique of DBS was first suggested by Dr. Robert Guthrie in the 1960s for diagnosis of phenylketonuria by neonatal screening [44]. In this sampling technique, a small volume of blood (30–50 *μ*L) is collected on filter paper or DBS cards based on a cellulose matrix with specific pore size and thickness [45, 46].

Several types of DBS cards are usually used for blood sampling in quantitative drug analysis. The FTA DMPK-A/ FTA card and FTA DMPK-B/ FTA elute card are coated and contain additives for analysis of nucleic acids [47]. Four types of DBS cards are uncoated: FTA DMPK-C/ 31ETF, Whatman 903, and two cards manufactured by Ahlstrom (226 and 237) [45]. After drying, a DBS sample is stored under conditions suitable for the analyte, usually at room temperature (RT) or 4°C. The sample could be sent by mail to a remote analytical laboratory if required.

A disk of a certain diameter is then punched and subjected to an extraction procedure. This procedure is simple: a suitable solvent is applied to dissolve the analyte in the punched disk of a DBS. Sometimes a second step, involving solid phase extraction (SPE) or liquid extraction (LLE), is required for further analysis [45]. The analysis involves a small amount of an analyte (as a consequence of the small volume of blood in a DBS): nanogram or even picogram amounts are suitable for fluorescence, MS, or MS/MS detection methods. Thus, the combination of the DBS technique with such methods allows for analyzing low concentrations of drugs in blood samples [30].

MS/MS includes selected-reaction monitoring (SRM) and multiple-reaction monitoring (MRM) modes, when only the transition between a selected precursor and product ions is detected. This approach enables excluding other possible ions and thus significantly improves sensitivity and selectivity of the assay [74].

At present, triple quadrupole (QQQ) mass-spectrometers remain the gold standard for quantification [74, 75], but quadrupole time-of-flight (QTOF) mass-spectrometers are also employed in many DBS studies.

Some analytical parameters are important for comparing assays of the same medication. First of all, there is the type of DBS card, size of the disk punched for analysis, and recovery of the analyte from a DBS; these directly affect the lower limit of quantitation (LLOQ). Assay sensitivity also depends on equipment type and detection mode. The extraction procedure with a suitable elution solvent is often characterized by simplicity. The linear concentration range and stability of DBS samples describe the assay in general.  Table 1 summarizes the methods of quantification of antihypertensive drugs in DBS, published from 2009 to 2017. The search for the articles was performed in the PubMed database by means of the following keywords: DBS or “dried blood spots” with the name of medications.

DBS sampling was reported for several classes of antihypertensive drugs, such as ACE inhibitors, ARAs, CCBs, alpha blockers, BBs, and ERAs as well as for statins. Although statins are not antihypertensive drugs themselves, they are often used as part of a combination therapy and enhance the antihypertensive effect of ACE inhibitors or CCBs, but not BBs or diuretics [76]. In most of the reviewed studies, statins have been simultaneously analyzed along with antihypertensive drugs.

## 3. ACE Inhibitors

These drugs block the peripheral conversion of angiotensin I to angiotensin II [77]. The DBS technique has predominantly been used by Tanna and coauthors for quantification of ramipril [48, 50, 51], captopril [53], and lisinopril [50]. Ramipril is the most studied medication among ACE inhibitors and is stable up to 84 days at RT with good recovery (~90%) from Whatman 903 and Ahlstrom 226 cards. Ramipril has been detected by QTOF mass-spectrometer with 417.2384 m/z. The only difference in the elution procedure was the extraction solvent: 300 *μ*L of methanol (MeOH) [50] or 150 *μ*L of MeOH/H_2_O (70:30, v/v) [48, 49, 51], and the latter showed better recovery. Lisinopril was analyzed in the same assay [50] in TOF mode at 406.2336 m/z. Captopril has been quantitated with similar results in SRM and TOF modes [53]. For this analysis, the Whatman 903 card was pretreated by dithiothreitol (DDT) and extraction solvent containing 10% v/v 200 mM DTT. It was necessary to reconstitute captopril from its disulfide dimer in blood samples. Ramiprilat is an active metabolite of ramipril and can be analyzed alone [52] or together with ramipril [51]. These studies cannot be completely compared because the assay was not described in detail in [51] and simply cited a paper where method of ramipril analysis was reported [48].

## 4. ARAs

ARAs specifically and selectively block type I angiotensin, a receptor of the renin-angiotensin system, by displacing angiotensin II from it [78, 79]. The presence of fluorescent functional groups in the molecular structure of ARA has allowed researchers to develop fluorescent assays for simultaneous quantification of valsartan, irbesartan, and losartan in DBS [54]. In this case, 1000 *μ*L of MeOH served as an extraction solvent with 98–99 % recovery. The assay sensitivity for valsartan was better than that reported in studies on MS detection in TOF mode [49, 50]. Irbesartan has been detected in MRM mode only as an internal standard (IS), but losartan detection in the MRM mode enables investigators to achieve LOQ of 1 ng/mL [55]. Losartan carboxylic acid is a more potent active metabolite of losartan [80], which has also been detected in MRM mode [55] with excellent recovery (93–98%). DBS samples of valsartan, irbesartan, losartan, and losartan carboxylic acid are stable up to 84, 30, 70, and 30 days at RT, respectively.

## 5. CCBs

Antihypertensive properties of CCBs are mediated by their ability to disrupt the movement of calcium ions through membrane channels. Several classes of CCBs can be distinguished based on their chemical structure: 1.4-dihydropyridines (amlodipine, pregabalin, and nifedipine), phenylalkylamine (verapamil), benzothiazepines (diltiazem), and others.

Verapamil has been used as a model analyte to develop and improve methods of analysis in DBS. For example, the following are direct assays via on-line desorption of DBS [58]: flow-through desorption of DBS without an LC column [57], the paper spray ambient ionization method for direct analysis of biological samples [59], and desorption electrospray ionization (DESI) that operates at atmospheric pressure [56].

In these works, verapamil has been detected by MS/MS with transitions 455 → 303 or 455 → 165 m/z. The paper spray method involving Whatman grade SG81 ion exchange paper shows the best sensitivity: down to 0.01 ng/mL. In this case, in contrast to other studies, the spot was cut out as a triangle (10 × 5 mm) [59]. Different solvents have been applied for elution: acetonitrile (ACN)/H_2_O, 100% ACN, and dichloromethane/isopropanol (90:10, v/v), but only for the flow-through desorption method was the recovery (81%) reported [57]. The stability of DBS samples in these studies has not been evaluated.

Amlodipine [49] and diltiazem [50] have been simultaneously analyzed along with ramipril and other medications under the conditions described above for ramipril.

To improve LOQ, the derivatization protocol with n-propyl chloroformate [81] has been developed for pregabalin quantitation in DBS [61]. The simple elution procedure with 0.1 M HCl yielded approximately 100% recovery. Nevertheless, LOQ was still high: 200 ng/mL.

The DBS technique has been applied to a study on the ways to increase stability of photosensitive compounds [60]. Nifedipine was used as a model analyte for this purpose. Extraction of nifedipine was performed on the Ahlstrom 226 card using micronic 96-well plates and silicone mats with a polytetrafluoroethylene film [60] by means of 200 *μ*L of ACN/H_2_O (70:30, v/v). It was shown that photosensitive compounds are more stable in DBSs than in blood or plasma under exposure to light.

## 6. Alpha Blockers

These are a class of antihypertensive drugs that has a vasodilating effect as a result of predominantly blocking *α*-adrenergic receptors. Guanfacine, like verapamil, has been chosen as a model analyte in several studies on improvement of DBS assays [62–64]. The analyte has been detected on the same equipment in SRM mode with transition 246.1 → 60.1 m/z. At first, the standard DBS technique was developed with good stability of DBS samples (76 days at RT), a simple extraction procedure (10 min mixing) involving 600 *μ*L of* tert*-butyl methyl ether (TBME), with ~80% recovery from a Whatman DMPK-C card and the concentration range from 0.05 to 25 ng/mL [62]. Then, methods of on-line desorption from a Whatman DMPK-C card [63] and from a two-layered polymeric membrane [64] were developed. The best sensitivity was achieved with the on-line DBS-SPE system based on Whatman DMPK-C cards. Doxazosin was simultaneously analyzed along with ramipril and other medications under the conditions described above for ramipril [49, 50]. Prazosin was analyzed by the DESI technique [56]. Several types of filter card were tested in that work: Whatman 903, FTA DMPK-A, FTA DMPK-B, and FTA DMPK-C. Nonetheless, the LOQ shown by the DESI technique was 100 ng/mL: not as low as in a comparable electrospray ionization assay [56].

## 7. BBs

This is a large group of drugs that block the binding sites on *β*-adrenergic receptors. Antihypertensive medications of this class are most often subjected to the DBS technique, for example, propranolol and bisoprolol. These two have been tested on different types of filter paper, including Sartorius TFN and Munktell TFN [66]. The DBS samples were stable on a Whatman 903 card for up to 30 and 84 days for propranolol [65] and bisoprolol [48, 49], respectively. The extraction procedure for bisoprolol was a standard one—mixing and sonication—whereas propranolol was extracted via on-line desorption [57] and paper heating [68]. The best recovery rates, up to 98–99%, for propranolol and bisoprolol were achieved with an extraction solvent consisting of MeOH or MeOH/H_2_O mixtures in different combination with or without 0.1% formic acid (FA). In contrast, extraction with 100 *μ*L of MeOH, 400 *μ*L of TBME, and 300 *μ*L of acetone resulted in 71% and 60% recovery for propranolol and bisoprolol, respectively [66]. The difference lies in the main detection mode: MRM for propranolol and TOF for bisoprolol. In two studies, atenolol was simultaneously assayed along with ramipril and other medications under conditions described above for ramipril [49, 50]. In the third paper, the DBS technique was generally the same and yielded similar results [29]. Metoprolol was extracted in two ways: by means of 100 *μ*L of MeOH, 400 *μ*L of TBME, and 300 *μ*L of acetone or with 200 *μ*L of 2% ammonium hydroxide and 700 *μ*L of ethyl acetate. The last solvent with simple 3 min mixing showed better recovery, up to 86%. In this case, the concentration range was 2.5–2500 ng/mL, with 14-day stability of DBS samples at RT.

## 8. ERAs

These drugs block endothelin receptors; ambrisentan acts on endothelin A receptors, whereas bosentan affects both endothelin A and B receptors. Only these two medications have been analyzed by the DBS sampling technique, and detection has been carried out in MRM mode for both. Elution of bosentan was performed using a Sample Card and Prep DBS System by means of MeOH/H_2_O (50:50, v/v) [70, 71] or simultaneously with ambrisentan in a standard assay with 400 *μ*L H_2_O and 4 mL of TBME [72]. The recovery in these two approaches was approximately the same, but bosentan recovery in the case of MeOH/H_2_O (50:50, v/v) was a little better. DBSs of both analytes were found to be stable for up to 147 days at RT.

## 9. Statins

These agents inhibit 3-hydroxy-3-methyl-glutaryl-coenzyme A reductase; as a result, the blood levels of total cholesterol, low-density lipoproteins, and triglycerides decrease, while the concentration of antiatherogenic high-density lipoproteins increases. The combination of statins with antihypertensive drugs increases the activity of the latter. Therefore, statins are often quantified simultaneously with antihypertensive drugs. Just as the analytes above, simvastatin [48–51] and atorvastatin [50] have been analyzed simultaneously with other medications under conditions described in a previous section for ramipril. The only problem with simvastatin is recovery, which did not exceed 68%. Rosuvastatin was assayed by 2-dimensional analytical-scale chromatography with at-column dilution enabling the injection of large sample volumes of organic extracts of DBS [73]. Analysis of rosuvastatin was performed in MRM mode with simple mixing extraction by means of 100 *μ*L of MeOH in the concentration range 5–80 ng/mL.

## 10. Effects of Blood Properties

Aside from the parameters of analysis described above, a researcher should take into account the effects of blood properties, such as hematocrit, blood volume, and blood distribution in a DBS as well as sample quality, which may have a direct impact on quantitative analysis. Hematocrit is the most important parameter influencing the accuracy and precision of DBS analysis [82, 83]. Hematocrit varies with gender, health status, and age and slightly with ethnicity [74]. Moreover, capillary blood tends to have higher hematocrit (e.g., 61%) than venous blood does [30]. Reference ranges slightly vary among sources but are typically 40–50% for adult men and 35–45% for adult women [30, 84, 85]. In a hospital population, 95% of routine hematocrit measurements yield values between 23% and 48% [74]. Hematocrit affects the spot size and formation, homogeneity of the spot, drying time, recovery of the analyte, and reproducibility and robustness of the assay [46, 74]. Therefore, it is important to evaluate the hematocrit effect during validation of an assay [46]. In several research papers, investigators have considered the influence of hematocrit on quantitative determination of analyte concentrations. No significant impact of hematocrit on the quantification was found in the hematocrit range 35–65% for bosentan [70], 38–45% for bosentan and ambrisentan [72], 23–43% for propranolol [65], 41–48% for guanfacine [62], and 20–50% for losartan and losartan carboxylic acid [55] and the hematocrit range of 35–55% for ramipril, lisinopril, valsartan, losartan, diltiazem, doxazosin, bisoprolol, atenolol, simvastatin, and atorvastatin [50]. The impact of hematocrit was evaluated in a follow-up to [64], where unacceptable bias was detected for guanfacine at hematocrit values of 30% and 60%, but acceptable accuracy and precision results were obtained at hematocrit 45% [86]. Besides, alternative approaches have been used for correction of hematocrit values: a mathematical equation for pregabalin [61], a number of consecutive analyses of the same blood spot for verapamil and propranolol [57], and analysis of the entire blood spot sample for ramiprilat, propranolol, and bisoprolol [52]. In some cases, the hematocrit effect was not studied because the authors assumed that hematocrit variation is likely to be within the “normal” hematocrit range in adults [29, 48]. In other studies, the hematocrit effect has not been considered.

Because it has been observed that the DBS area decreases nearly linearly when hematocrit increases, the spot size and homogeneity of spots also influence quantification results [74, 84]. Usually, the spotted blood volume is between 15 and 40 *μ*L. Accordingly, the impact of the blood spotting volume on DBS analysis has been investigated in several works: blood volume of 20, 25, and 30 *μ*L [70]; 20, 30, and 40 *μ*L [29, 48, 50, 53]; 10 to 40 *μ*L [69]; 15, 20, and 25 *μ*L [62]; and 10, 15, and 20 *μ*L [55]. In all cases, accuracy and precision were less than 15% within the tested range, and therefore accurate pipetting during preparation of DBS may not be necessary. To minimize the influence of spot homogeneity on measurement, the same location of a spot (center) [29, 48–51, 53, 54, 61–63, 69, 73] or the whole spot [52, 66] has been punched. No difference between punching the central area and punching an off­center site of DBS has been observed for pregabalin [61] and guanfacine [62].

In many cases, analyte concentration can differ between capillary and venous blood [87] as well as between plasma and DBS. Thus, a comparison is recommended as part of the assay validation [30]. Such a study has been conducted for guanfacine, showing on average 20% higher concentrations in DBS than in plasma, but an 11.6% to 23.2% decrease in concentration was observed when whole blood rather than plasma was spiked with guanfacine [62]. The analyte concentrations in plasma show a significant correlation with DBS concentrations with a conversion factor (slope) of 1.73 for propranolol [65], 1.58 for ambrisentan, and 1.52 for bosentan [72]. Bosentan DBS and plasma concentrations have also been compared in a Bland–Altman plot, and a limited systematic difference between the measurements was found [71]. For rosuvastatin, quantification methods for either plasma or DBS have been developed, but comparison experiments for plasma and DBS samples have not been carried out [73]. For nifedipine, only photostability has been compared between DBS and liquid aqueous and biological matrices [60].

Samples during validation assays are collected in the laboratory. If samples are collected in the laboratory from volunteers [29, 48–51] or rats [52, 54], they are usually spotted correctly. For home sample collection, the proportion of unsatisfactory samples is 19% [49]. This figure can reach more than 30% [74, 88] and may pose a problem for clinical application because the assays are validated by a correct technique for spotting of samples.

## 11. Conclusions

Interest in the DBS technique as an easy blood sampling method for monitoring of antihypertensive drug concentrations is increasing continuously. The main limitation of the DBS technique is sensitivity, which is expected to improve with the growing availability of MS and MS/MS equipment in clinical and scientific laboratories for analysis of antihypertensive drugs. Among the reviewed works, medications have in general been analyzed in MRM mode on QQQ mass-spectrometers or in TOF mode on QTOF mass-spectrometers. The elution procedure in many cases consists of mixing and sonication in an extraction solvent with suitable recovery. Most popular types of filter paper are Whatman 903 or Whatman DMPK-C/-A cards. Almost all antihypertensive drugs show great stability in DBS samples for weeks or even months. An automated DBS technique and on-line desorption of DBS have been devised in some studies to enable analysis of a large number of samples. The major variables of the DBS technique are hematocrit and differences in drug concentration measured in blood, blood plasma, and DBS. Validation experiments are necessary for new DBS assays, but not all the published drug assays involving DBS include full validation; this situation can be considered the main obstacle for their broad application.

## Figures and Tables

**Table 1:** Published DBS sampling techniques for analysis of antihypertensive medications and statins.

Drug analyzed	DBS card/paper used	Diameter of disk punched for analysis, mm	Calibration range, ng/mL	Stability of DBS samples	Elution procedure (min)	V, Extraction solvent	Recovery (%)	Mass-spectrometer	Detection mode	Mass or transmission, m/z	Internal standard	Ref.
**Angiotensin converting enzyme inhibitors**												

Ramipril	Whatman 903	8	0.5 - 100	84 days, RT	mixed (1 min) + sonicated (30 min)	150 *μ*L, MeOH/H_2_O (70:30, v/v)	≥92	Agilent G6530A QTOF	TOF	417.2384	Atenolol	[48]
	Whatman 903	8	-	84 days, RT	mixed (1 min) + sonicated (30 min)	150 *μ*L, MeOH/H_2_O (70:30, v/v)	-	Agilent 6530 QTOF	TOF	417.2384	Atenolol	[49]
	Whatman 903	8	0.1 - 100	70 days, RT	mixed (1 min) + sonicated (30 min)	300 *μ*L, MeOH	≥87	Agilent G6530A QTOF	TOF	417.2384	Atenolol-d7	[50]
	Ahlstrom 226	8	See [48]	-	mixed (1 min) + sonicated (30 min)	150 *μ*L, MeOH/H_2_O (70:30, v/v)	See [48]	Agilent G6530A QTOF	TOF	417.2384	Atenolol	[51]

Ramiprilat	Whatman 903	Whole DBS	5 - 250	21 days, 25°C 21 days, 37°C 21 days,-20°C	mixed (1 min) + sonicated (10 min) + mixed (45 min)	300 *μ*L, MeOH/0.1% FA (90:10, v/v)	65-81	Agilent 6460 QQQ	MRM	389.2 → 206.1	Ramiprilat-d5	[52]
	Ahlstrom 226	8	See [48]	-	mixed (1 min) + sonicated (30 min)	150 *μ*L, MeOH/H_2_O (70:30, v/v)	See [48]	Agilent G6530A QTOF	TOF	389.2071	Atenolol	[51]

Captopril	Whatman 903	8	10 - 400	84 days, RT	mixed (1 min) + sonicated (30 min)	200 *μ*L, MeOH/H_2_O (60:40, v/v) with 10% v/v 200 mM DTT	90 ± 10	Agilent 1100 Ion Trap Agilent 6530 TOF	SRM TOF	218.0 218.0845	Enalapril maleate	[53]

Lisinopril	Whatman 903	8	0.1 - 100	70 days, RT	mixed (1 min) + sonicated (30 min)	300 *μ*L, MeOH	≥87	Agilent G6530A QTOF	TOF	406.2336	Atenolol-d7	[50]

**Angiotensin II receptor antagonists**												

Valsartan	Whatman 903	8	50 - 4000	70 days, RT	mixed (1 min) + sonicated (30 min)	300 *μ*L MeOH	≥87	Agilent G6530A QTOF	TOF	436.2343	Atenolol-d7	[50]
	Whatman 903	8	-	84 days, RT	mixed (1 min) + sonicated (30 min)	150 *μ*L, MeOH/H_2_O (70:30, v/v)	-	Agilent 6530 QTOF	TOF	436.2343	Atenolol	[49]
	Whatman DMPK-A	10	6 - 2000	30 days, RT	mixed (15 min)	1000 *μ*L, MeOH	98	-	fluorescence	-	-	[54]

Irbesartan	Whatman 903	3	20	30 days, RT	mixed (5 min) + sonicated (10 min)	500 *μ*L, MeOH/H_2_O (50:50, v/v)		Agilent 1100 Ion Trap	MRM	427.3 → 193.0	Irbesartan	[55]
	Whatman DMPK-A	10	6 - 2000	30 days, RT	mixed (15 min)	1000 *μ*L, MeOH	99	-	fluorescence	-	-	[54]

Losartan	Whatman 903	8	5 - 1000	70 days, RT	mixed (1 min) + sonicated (30 min)	300 *μ*L, MeOH	≥87	Agilent G6530A QTOF	TOF	423.1695	Atenolol-d7	[50]
	Whatman 903	3	1 - 200	30 days, RT	mixed (5 min) + sonicated (10 min)	500 *μ*L, MeOH/H_2_O (50:50, v/v)	89-94	Agilent 1100 Ion Trap	MRM	421.2 → 179.0	Irbesartan	[55]
	Whatman DMPK-A	10	12 - 4000	30 days, RT	mixed (15 min)	1000 *μ*L, MeOH	98	-	fluorescence	-	-	[54]

Losartan carboxylic acid	Whatman 903	3	5 - 1000	30 days, RT	mixed (5 min) + sonicated (10 min)	500 *μ*L, MeOH/H_2_O (50:50, v/v)	93-98	Agilent 1100 Ion Trap	MRM	421.2 → 179.0	Irbesartan	[55]

**Calcium channel blockers**												

Verapamil	Whatman 903 Whatman DMPK-C Ahlstrom 237 Whatman DMPK-A Whatman DMPK-B	3	500		mixed (60 min)	200 *μ*L, ACN/H_2_O (75:25, v/v)		AB Sciex API 4000	SRM	455 → 165	Verapamil	[56]
	Whatman DMPK-C	2 *∗*	1 - 1000		Used SPE cartridge	1000 *μ*L, 0.2% FA in H_2_O	81	AB Sciex API 4000	MRM	455.3→ 165.2	Imipramine	[57]
	Whatman filter paper, Grade CF 12	Whole DBS	50 - 500	-	On-line desorption	100% ACN	-	Agilent 1100 MSD single Q VL version	SRM	-	Trimipramine-d3	[58]
	Whatman grade SG81 ion exchange paper	Whole DBS	0.01 - 10000	-	Paper spray	MeCl_2_/isopropanol (90:10, v/v)	-	Thermo TSQ Quantum Access Max QQQ	SRM	455 → 303	-	[59]

Amlodipine	Whatman 903	8	-	84 days, RT	mixed (1 min) + sonicated (30 min)	150 *μ*L, MeOH/H_2_O (70:30, v/v)	-	Agilent 6530 QTOF	TOF	431.1344	Atenolol	[49]

Nifedipine	Ahlstrom 226	4	10 - 10000	1 day, RT	-	200 *μ*L, ACN/H_2_O (75:25, v/v)	-	AB Sciex API 4000	MRM	347→ 315	[^13^C,^15^N_2_]-nifedipine nitrophenyl pyridine	[60]

Pregabalin	Whatman 903	4	200 - 20000	120 days, 4°C	mixed (10 min) + Derivatization	100 *μ*L, 0.1 mol/L HCl	98-102	Thermo TSQ Quantum Access MAX QQQ	SRM	288.0 → 142.1 288.0 → 228.0	4-aminocyclohexanecarboxylic acid	[61]

Diltiazem	Whatman 903	8	0.5 - 600	70 days, RT	mixed (1 min) + sonicated (30 min)	300 *μ*L, MeOH	≥87	Agilent G6530A QTOF	TOF	415.1686	Atenolol-d7	[50]

**α** ** blockers**												

Guanfacine	Whatman DMPK-C	5	0.05 - 25	76 days, RT	mixed (10 min)	600 *μ*L, TBME	~80	AB SCIEX 5500 QTRAP	SRM	246.1→ 60.1	[^13^C,^15^N_3_]-guanfacine hydrochloride	[62]
	Whatman DMPK-C	4 *∗*	0.01 - 25	-	On-line DBS-SPE system	1000 *μ*L, ACN/0.1% FA (80:20, v/v) + 1000 *μ*L, 0.1% FA	-	AB SCIEX 5500 QTRAP	SRM	246.1 → 60.1	[^13^C,^15^N_3_]-guanfacine hydrochloride	[63]
	two-layered polymeric membrane	2 *∗*	0.25 - 250	-	On-line DBS-SPE system	1000 *μ*L, ACN/0.1% FA (80:20, v/v) + 1000 *μ*L, 0.1% FA	-	AB SCIEX 5500 QTRAP	SRM	246.1 → 60.1	[^13^C,^15^N_3_]-guanfacine hydrochloride	[64]

Doxazosin	Whatman 903	8	-	84 days, RT	mixed (1 min) + sonicated (30 min)	150 *μ*L, MeOH/H_2_O (70:30, v/v)	-	Agilent 6530 QTOF	TOF	452.1928	Atenolol	[49]
	Whatman 903	8	0.1 - 100	70 days, RT	mixed (1 min) + sonicated (30 min)	300 *μ*L, MeOH	≥87	Agilent G6530A QTOF	TOF	452.1928	Atenolol-d7	[50]

Prazosin	Whatman 903 Whatman DMPK-C Ahlstrom 237 Whatman DMPK-A Whatman DMPK-B	3	100 - 10000	-	mixed (60 min) + sonicated (30 min)	200 *μ*L, ACN/H_2_O (75:25, v/v) or MeOH for DMPK-B	-	AB Sciex API 4000	SRM	384 → 247	Verapamil	[56]

**β** ** blockers**												

Propranolol	Whatman 903	Whole DBS	5 - 250	21 days, 25°C 21 days, 37°C 21 days,-20°C	mixed (1 min) + sonicated (10 min)+ mixed (45 min)	300 *μ*L, MeOH/0.1% FA (90:10, v/v)	91-99	Agilent 6460 QQQ	MRM	260.2 → 116.1	Propranolol-d7	[52]
	Whatman 903	3.2	2.5 - 200	30 days, RT 30 days, 4°C 30 days,-20°C	mixed (25 min)	200 *μ*L, MeOH/0.1% FA (95:5, v/v)	94-100	AB SCIEX 5500 QTRAP	MRM	260.1 → 116.1	Propranolol-d7	[65]
	Whatman DMPK-C	2 *∗*	1 - 1000	-	Used SPE cartridge	1000 *μ*L, 0.2% FA in H_2_O	83	AB Sciex API 4000	MRM	260.1→ 116.1	Imipramine	[57]
	Sartorius TFN, Munktell TFN, Whatman DMPK-C	Whole DBS	0 - 20	7 days, 4°C	sonicated (45 + 30 min)	100 *μ*L, MeOH + 400 *μ*L, TBME + 300 *μ*L, acetone	71	Thermo Q Exactive	scan-to-scan	260.1645	Cocaine-d3	[66]
	Watman DMPK-B	3	1 - 500	-	mixed (10 min)	300 *μ*L, MeOH	-	AB Sciex API 4000	MRM	-	Propranolol-d3	[67]
	Whatman filter paper	4 *∗*	1 - 500	-	Heating	25 *μ*L, MeOH with 0.1% FA	70–82	AB SCIEX 3200 QTRAP	MRM	260 → 183	Propranolol-d7	[68]

Bisoprolol	Whatman 903	8	0.1 - 100	70 days, RT	mixed (1 min) + sonicated (30 min)	300 *μ*L, MeOH	≥87	Agilent G6530A QTOF	TOF	326.2326	Atenolol-d7	[50]
	Whatman 903	8	0.1 - 100	84 days, RT	mixed (1 min) + sonicated (30 min)	150 *μ*L, MeOH/H_2_O (70:30, v/v)	≥92	Agilent G6530A QTOF	TOF	326.2326	Atenolol	[48]
	Whatman 903	8	-	84 days, RT	mixed (1 min) + sonicated (30 min)	150 *μ*L, MeOH/H_2_O (70:30, v/v)	~98	Agilent 6530 QTOF	TOF	326.2326	Atenolol	[49]
	Whatman 903	Whole DBS	5 - 250	21 days, 25°C 21 days, 37°C 21 days,-20°C	mixed (1 min) + sonicated (10 min)+ mixed (45 min)	300 *μ*L, MeOH/0.1% FA (90:10, v/v)	74-83	Agilent 6460 QQQ	MRM	326.2 → 116.1	Bisoprolol-d5	[52]
	Sartorius TFN, Munktell TFN, Whatman DMPK-C	Whole DBS	0 - 20	7 days, 4°C	sonicated (45 + 30 min)	100 *μ*L, MeOH + 400 *μ*L, TBME + 300 *μ*L, acetone	60	Thermo Q Exactive	scan-to-scan	326.2326	Cocaine-d3	[66]
	Ahlstrom 226	8	See [48]	See [48]	mixed (1 min) + sonicated (30 min)	150 *μ*L, MeOH/H_2_O (70:30, v/v)	See [48]	Agilent G6530A QTOF	TOF	326.2326	Atenolol	[51]

Atenolol	Whatman 903	8	-	84 days, RT	mixed (1 min) + sonicated (30 min)	150 *μ*L, MeOH/H_2_O (70:30, v/v)	-	Agilent 6530 QTOF	TOF	267.1703	Atenolol	[49]
	Whatman 903	8	10 - 1500	70 days, RT	mixed (1 min) + sonicated (30 min)	300 *μ*L, MeOH	≥87	Agilent G6530A QTOF	TOF	267.1703	Atenolol-d7	[50]
	Whatman 903	5	25 - 1500	70 days, 25°C	mixed (1 min) + sonicated (30 min)	200 *μ*L, MeOH/H_2_O (60:40, v/v)	96-100	Agilent 6530 QTOF	TOF	267.1703	Atenolol-d7	[29]

Metoprolol	Whatman DMPK-A	5	2.5 - 2500	14 days, RT	mixed (3 min)	200 *μ*L, 2% ammonium hydroxide + 700 *μ*L, ethyl acetate	77-86	AB Sciex API 5000	MRM	268.2 → 116.2	Metoprolol-d7	[69]
	Sartorius TFN, Munktell TFN, Whatman DMPK-C	Whole DBS	0 - 20	7 days, 4°C	sonicated (45 + 30 min)	100 *μ*L, MeOH + 400 *μ*L, TBME + 300 *μ*L, acetone	68	Thermo Q Exactive	scan-to-scan	268.1907	Cocaine-d3	[66]

**Endothelin receptor antagonists **												

Bosentan	Whatman DMPK-A	-	2 - 1500	105 days, RT	Used Sample Card And Prep DBS System	MeOH/H_2_O (50:50, v/v)	83-92	AB Sciex API 4000	MRM	552.2 → 202.1	Bosentan-d4	[70]
	Whatman DMPK-A	-	2 - 3000	105 days, RT	Used Sample Card And Prep DBS System	MeOH/H_2_O (50:50, v/v)	83-92	AB Sciex API 4000	MRM	552.2 → 202.1	Bosentan-d4	[71]
	Whatman DMPK-C	6.2	2.5 - 4000	147 days, RT 14 days, 37°C 14 days,-20°C	sonicated (20 min) + mixed (20 min)	400 *μ*L, H_2_O + 4000 *μ*L, TBME	69-83	Thermo TSQ7000 QQQ	MRM	552.4 → 202.1	Bosentan-d4	[72]

Ambrisentan	Whatman DMPK-C	6.2	2.5 - 1000	147 days, RT 14 days, 37°C 14 days,-20°C	sonicated (20 min) + mixed (20 min)	400 *μ*L, H_2_O + 4000 *μ*L, TBME	88-94	Thermo TSQ7000 QQQ	MRM	347.3 → 125	Ambrisentan-d10	[72]

**Statins**												

Simvastatin	Whatman 903	8	1 - 100	84 days, RT	mixed (1 min) + sonicated (30 min)	150 *μ*L, MeOH/H_2_O (70:30, v/v)	~43	Agilent G6530A QTOF	TOF	441.261 Na adduct	Atenolol	[48]
	Whatman 903	8	0.1 - 100	70 days, RT	mixed (1 min) + sonicated (30 min)	300 *μ*L, MeOH	68	Agilent G6530A QTOF	TOF	441.2611	Atenolol-d7	[50]
	Whatman 903	8	-	84 days, RT	mixed (1 min) + sonicated (30 min)	150 *μ*L, MeOH/H_2_O (70:30, v/v)	~40	Agilent 6530 QTOF	TOF	441.2611	Atenolol	[49]
	Ahlstrom 226	8	See [70]	See [70]	mixed (1 min) + sonicated (30 min)	150 *μ*L, MeOH/H_2_O (70:30, v/v)	See [70]	Agilent G6530A QTOF	TOF	418.2719 / 441.2611 NA adduct	Atenolol	[51]

Atorvastatin	Whatman 903	8	0.5 - 100	70 days, RT	mixed (1 min) + sonicated (30 min)	300 *μ*L, MeOH	≥87	Agilent G6530A QTOF	TOF	559.2610	Atenolol-d7	[50]

Rosuvastatin	Whatman DMPK- B	3	0.5 - 80		mixed (60 min)	100 *μ*L, MeOH		Waters Xevo TQ-S QQQ	MRM	482 → 258	Rosuvastatin-d6	[73]

ACN: acetonitrile, FA: formic acid, DTT: dithiothreitol, MeCl_2_: dichloromethane, MeOH: methanol, MSX: multiplex targeted SRM mode, and TBME: *tert*-butyl methyl ether.

*∗*: elution area
